# Optimizing Electronic Consultation Between Primary Care Providers and Psychiatrists: Mixed-Methods Study

**DOI:** 10.2196/jmir.8943

**Published:** 2018-04-06

**Authors:** Jennifer M Hensel, Rebecca Yang, Minnie Rai, Valerie H Taylor

**Affiliations:** ^1^ Women's College Hospital Institute for Healthcare Solutions and Virtual Care Women's College Hospital Toronto, ON Canada; ^2^ Department of Psychiatry Women's College Hospital Toronto, ON Canada; ^3^ Department of Psychiatry University of Toronto Toronto, ON Canada

**Keywords:** eHealth, psychiatry, primary care, consultation, health services

## Abstract

**Background:**

The use of electronic consultation (e-consult) between primary care providers (PCPs) and psychiatrists has potential, given the high prevalence of mental health issues in primary care and problematic access to specialist care. Utilization and uptake, however, appears to be lower than would be expected.

**Objective:**

This study aimed to examine actual utilization of e-consult between PCPs and psychiatrists and investigate the perceptions of PCPs about this form of psychiatric advice to inform how to optimize the utility and thereby the uptake of this service.

**Methods:**

In this mixed-methods study, we conducted a chart review of psychiatry e-consults (N=37) over 2 platforms during early implementation in Ontario, Canada, as well as 3 group interviews and 1 individual interview with PCPs (N=10) with variable experience levels and from a range of practice settings. The chart review assessed response times and referral content including the type of request, referral attachments, and consultant responses. Interviews explored the perceptions of the PCPs about the uses and barriers of psychiatry e-consult. Thematic content analysis of interview data identified common themes as well as themes unique to different provider profiles (eg, experienced PCPs vs new PCPs and rural vs urban practice). On the basis of interpretation of the quantitative and qualitative findings, we developed recommendations for the optimization of psychiatry e-consultation services.

**Results:**

During the study period, psychiatry e-consults comprised 3.66% (49/1339) of all e-consults submitted on the studied platforms. Among the e-consults reviewed, different psychiatric diagnoses were represented: 70% of requests (26/37) queried about medication safety or side effects, whereas 59% (22/37) asked about psychiatric symptom management. Moreover, 81% (30/37) of e-consults were answered within 24 hours, and 65% (24/37) were addressed in a single exchange. Themes from the interview data included psychiatry having a complexity that differentiates it from other specialties and may limit the utility of e-consult, other than for psychopharmacology advice. Variability in awareness exists in the way e-consultation could be used in psychiatry, with new PCPs feeling unsure about the appropriateness of a question. In general, new PCPs and PCPs practicing in rural areas were more receptive to psychiatry e-consult. PCPs viewed e-consult as an opportunity to collaborate and desired that it be integrated with other available services. Recommendations include the need for appropriate specialist staffing to address a wide range of requests, adequate education to referrers regarding the use of psychiatry e-consult, and the need to integrate psychiatry e-consult with other geographically relevant services, given the complexity of psychiatric issues.

**Conclusions:**

E-consult is a viable and timely way for PCPs to get much-needed psychiatric advice. For optimizing its utility and uptake, e-consult needs to be integrated into reliable care pathways with adequate referrer and consultant preparation.

## Introduction

Specialist wait times are a major health care access barrier [1-3]. As a potential solution to specialist wait times, electronic consultations (e-consults) were used to facilitate rapid access to specialist advice via asynchronous written communication [4]. Data across a range of medical specialties support that 75% of e-consults receive a timely response within 3 days, and most take less than 10 min to complete, with up to one third of intended in-person referrals being avoided [5]. Primary care providers (PCPs) are extremely satisfied with e-consult due to its convenience and its contribution to their confidence in managing their patients [5,6].

E-consult between PCPs and psychiatrists has potential as some of the longest specialist wait times have been observed in psychiatry [7]. The studies, which simulated psychiatric referrals from primary care in Canadian and US cities, confirmed that referrals were frequently rejected altogether [8,9]. This would be highly problematic as mental illness is among the leading causes of disability, with economic implications projected to rise by five or six times in the next 30 years [10,11]. As such, rapid access to advice from a psychiatrist would be extremely beneficial for PCPs and their patients. Recently published studies have reported that PCPs feel increased support as a result of having access to psychiatry e-consult [12], and that psychiatry e-consults are predominantly used to address medication-related questions. Utilization of e-consult for psychiatry has been low, however, relative to other specialties [6,13] and the theoretical need that might exist based on mental health visit volumes to primary care [14,15]. The aim of this study was to quantitatively examine actual utilization and the content of e-consults between PCPs and psychiatrists and qualitatively investigate the perceptions of PCPs about this form of psychiatric advice, to inform how to optimize the utility and thereby the uptake of this service as a mechanism for consultation.

## Methods

### Study Design

We used a convergent parallel mixed-methods study design consisting of a retrospective chart review of completed e-consults and a series of interviews with PCPs. The overall approach to the study was a pragmatic one, seeking practical solutions for programmatic change that triangulated between the quantitative and qualitative results, as well as the existing literature [16]. Quantitative and qualitative analyses were completed separately and integrated, looking for both convergence and divergence of findings [17] to inform recommendations. Research ethics approval was obtained from the Women’s College Hospital Research Ethics Board.

### Chart Review

#### Setting and Participants

A sample of e-consults completed between January 1, 2015, and March 31, 2016, was obtained from 2 e-consult platforms (one private and one government-funded) available in Ontario, Canada. These platforms were implemented in target regions and have gradually expanded to be accessible to most of the province with voluntary participation. Platforms offer PCPs access to a range of specialists, with compensation for consultants and referrers. The operator of each platform provided the study team with utilization data for the study period and email addresses for psychiatrist consultants who were requested to provide their e-consultation for review. There are no restrictions for referrers regarding the types of questions that can be posed over these e-consult platforms, although psychiatrists may specify their areas of expertise.

#### Data Collection and Analysis

Among the 8 active psychiatrists, 5 provided all of their de-identified e-consult reports to the study team by secure fax or encrypted email. All the consultations were reviewed to capture the content using a data collection form, which was modified iteratively during the chart review until a set of nonexclusive categories were established. Data collected included referrer details and patient’s age, gender, and psychiatric diagnosis. We classified the content of the referral question, types of referral attachments, and the components of the consultant’s response including any attachments provided back to the referrer, and calculated time to consultant response and the number of exchanges. Data were inputted into a Microsoft Access database and extracted into Microsoft Excel for analysis. From the data, we generated variable counts and means, where applicable.

### Qualitative Interviews

#### Setting and Participants

We conducted 4 interviews with a total of 10 PCPs—3 were small group interviews and 1 was an individual interview. We used purposive and maximal variation sampling to recruit participants of different experience levels, such as PCPs from urban and rural areas, and those conducting group and solo practice. Details of the interviewed participants are presented in Table 1.

#### Data Collection and Analysis

Interviews were conducted by one of the 2 researchers who had no pre-existing relationship with the participants. Participants were asked about their previous experience with e-consult and 3 questions specifically about e-consult for psychiatry:

1. Do you think e-consult is useful for psychiatry?
2. What are the limitations or barriers of using e-consult for psychiatry?
3. Is there anything that would make e-consult more useful for psychiatry?

**Table 1:** Focus group participants. N/A: not applicable.

Interview	Participants	Practice location	Type of practice	Years in practice, mean (range)	Type of interview	Length of interview (min)
1	5 family physicians	Urban	Group-based^a^	17.8 (10-36)	In-person (at a clinic meeting)	30
2	1 family physician	Urban	Solo practice	3 (N/A)	Teleconference	10
3	2 family physicians	Urban	1 solo practice; 1 group-based	2.5 (2-3)	Teleconference	15
4	1 family physician and 1 nurse practitioner	Rural	Solo practice	6.5 (5-8)	Teleconference	20

^a^Refers to a group-based practice of family physicians and nurse practitioners with access to multidisciplinary support including social work and colocated psychiatry with wait times till on the order of months.

Open-ended questions were used intentionally to generate discussions between the participants. Interviews varied in length from 10 to 30 min, and were audio-recorded and transcribed verbatim. Subsequently, 2 reviewers independently openly coded each transcript, and then convened to compare codes. From the first transcript of a group interview, preliminary themes were developed, and a constant comparative analysis was applied for subsequent transcripts. The coders reviewed for common themes overall, as well as common and contrasting themes from different participant profiles (eg, experienced vs novice and urban vs rural). The reviewers also applied intersubjectivity during coding and thematic development [16], drawing from both the objective data and their subjective personal experience, as one was an e-consult psychiatrist (JH) and the other had been involved in local e-consult implementation (RY). Once a final set of themes was agreed upon, transcripts were independently rereviewed, and any disagreements were discussed until consensus was reached in all cases.

## Results

### Chart Review

Between January 1, 2015, and March 31, 2016, among all e-consults submitted across all specialty areas, 3.66% (49/1339) e-consults were submitted for psychiatry on the 2 platforms: 4.2% (38/887) on the government-funded platform and 2.4% (11/452) on the private platform. Out of the psychiatric e-consults, 78% (38/49) were obtained from the psychiatrists for review. Of them, one e-consult had been declined and resubmitted to another psychiatrist; thus, it was counted only once. The e-consults represented 37 different patients from 30 unique PCP referrers (ranging from 1 to 5 e-consults each). The 5 psychiatrists answered between 1 and 25 e-consults each. The consultant knew the patient from a previous encounter in only one of the cases. Table 2 displays referral characteristics. Most requests (86%, 32/37) were submitted by family physicians, with 14% (5/37) coming from nurse practitioners. The years of practice of the referrers ranged from 1 to 42 years. Patients were mostly female (65%, 24/37), ranging in age from 15 to 90 years. A diverse range of diagnoses were represented, with more than half (57%, 21/37) of referrals having two or more diagnoses documented. The most common referral question was about medication side effects or safety (70%, 26/37), followed by psychiatric symptom management (59%, 22/37; Table 3). Most questions about medication side effects or safety pertained to antidepressants, followed by antipsychotics, lithium, stimulants, and benzodiazepines. Questions about side effects or safety can be clustered into 3 main groups: (1) medical complications (eg, weight gain, hypothyroidism, sexual dysfunction), (2) safety in special populations (eg, pregnant, elderly, pediatric), and (3) psychiatric complications (eg, manic switch, suicidal ideation). Referrers provided various attachments for the specialist to review (Table 3).

Most e-consults (81%, 30/37) were responded to within 24 hours and nearly two-thirds (65%, 24/37) were answered with a single exchange between referrer and consultant (Table 3). Among the 8 cases where the consultant suggested a referral to a specialist for assessment, 5 were for psychiatry, 2 for cognitive behavioral therapy, and 1 for endocrinology. Consultants frequently suggested or attached provider and patient resources, which often included e-resources (Table 3).

### Qualitative Interviews

Previous experience with e-consult among interviewed PCPs varied from minimal to frequent use. Participants described preferred general conditions for the use of e-consult such as “quick,” “simple” questions to a consultant who is reliable in responding promptly. They also discussed the preference for e-consult to be integrated with electronic medical records for ease of use, and a desire for short, unstructured forms. Several themes specific to perceptions regarding e-consult for psychiatry were identified.

#### Psychiatry is Perceived as More “Nuanced” Than Other Specialties Limiting Electronic Consultation Uses Other Than Psychopharmacology Advice

PCPs felt that e-consults were more applicable in other medical specialties where consultants could advise on objective assessments, compared with psychiatry, where the questions were pertinent to the entire person and often too “nuanced” for e-consult:

Most times when I use e-consults in general, it’s usually for an abnormal result, an abnormal lab, an abnormal ultrasound, that I’m debating how urgent it is, what’s the next step, is this a formal consultation. That’s really not the situation in psychiatry, where anything that I would get abnormal, related to psychiatry, I can manage...It’s really the nuances in the interaction...

Interview 1

**Table 2:** Referrer and patient characteristics from e-consults (N=37).

Referrer or patient characteristic		Value
Referrer years of practice, mean (range)		14.9 (1-42)
**Referrer discipline, n (%)**		
	Family physician	32 (86)
	Nurse practitioner	5 (14)
**Patient gender, n (%)**		
	Male	13 (35)
	Female	24 (65)
Patient age, mean (range)		39.7 (15-90)
**Patient diagnosis, n (%)**		
	Depression	17 (46)
	Anxiety	16 (43)
	Bipolar disorder	6 (16)
	Posttraumatic stress disorder	4 (11)
	Substance use disorder	4 (11)
	Attention deficit hyperactivity disorder	4 (11)
	Sleep disorder	3 (8)
	Psychotic disorder	2 (5)
	Obsessive compulsive and related disorders	2 (5)
	Intellectual and developmental disability	1 (3)
	Other	3 (8)
	Not stated	2 (5)
	Two or more diagnoses	21 (57)

A participant stated, “psych(iatry) is so messy” (Interview 3). Moreover, as PCPs frequently provide mental health care, they are “pretty comfortable dealing with frontline psychiatric issues” (Interview 1). In these cases, they feel “it’s rare, in fact, that [they] need to consult with psychiatry at all” (Interview 1). During the first group interview, comprising experienced PCPs, a strong group consensus was observed on this perspective. When the PCPs felt the need to consult, they expressed a preference for shared assessment and management of these complex cases:

Often when I need to refer somebody to psych(iatry), it’s a pretty complicated issue where I feel like they actually need to see the person and it’s not something that I can convey via [e-consult].

Interview 3

However, although all participants referred to the nuances of the psychiatric patient, they also identified a particular use of e-consult for medication advice. In this context, a participant stated:

But I just think...you need to see them,...Unless it’s something like medication optimization and things like that.

Interview 3

Examples of using e-consult included “ensuring (medication) safety in pregnancy” (Interview 1) and getting assistance with the application of treatment guidelines in unique situations:

There’s a lot of guideline support that’s out there, which certainly I can rely on guideline support and not use the e-consult. But typically, the guideline support doesn’t come with the vast experience that e-consult psychiatrists do.

Interview 4

A dedicated psychopharmacology e-consult service was suggested rather than an unspecified service to ensure that the PCP seeks relevant advice from a knowledgeable specialist.

#### A Lack of Awareness on the Range of Uses for Psychiatric Electronic Consultation Impacts Its Utilization and Perceived Utility

The first interview exposed variable awareness regarding the use of e-consult. A PCP mentioned using an e-consult to help with resource navigation for a patient, whereas another participant found the suggestion given via e-consult to be very compelling, and referred to it several times throughout the interview.

Moreover, another PCP described the shift in perspective after understanding the use of a psychiatry e-consult:

I think when it was first presented to me I was a little skeptical. When I saw some of the use cases it was definitely helpful.

Interview 2

**Table 3:** Content and outcome of e-consult (N=37).

Referral domain			Value, n (%)
**Content**			
	**Reason for referral^a^**		
		Medication side effects or safety^b^	26 (70)
		Psychiatric symptom management	22 (59)
		Role of co-occurring medical illness	9 (24)
		Seeking behavioral intervention strategies	1 (3)
	**Referral attachments**		
		Typed consult note	9 (24)
		Cumulative patient profile	7 (19)
		Previous consult reports	6 (16)
		Laboratory results	4 (11)
		Photos	1 (3)
**Outcome**			
	**Time to respond**		
		Within 24 hours	30 (81)
		Between 24 and 72 hours	5 (14)
		More than 72 hours	2 (5)
	**Number of exchanges^c^**		
		One	24 (65)
		Two	12 (32)
		More than two	1 (3)
	**Consultant response**		
		Requested clarification about question	9 (24)
		Suggested referral	8 (22)
		Attached provider resources (including e-resources)	14 (38)
		Attached patient resources (including e-resources)	8 (22)

^a^Referrals may have contained questions that fit into more than one classification.

^b^Medication side effects/safety includes medical complications such as weight gain, hypothyroidism, and sexual dysfunction (n=10); safety in special populations, such as pregnant, pediatric, and elderly (n=9); psychiatric complications, such as antidepressant-induced mania or suicidal ideation (n=3); and other (n=6).

^c^An exchange includes one message from each of the referrer and consultant related to the referral question. If a reply only expressed gratitude for information provided, it was not counted.

New PCPs expressed more uncertainty regarding the appropriateness of a question to the specialist than more experienced PCPs:

I’m worried that I’m bothering the psychiatrist, or it’s not an appropriate question, maybe a little bit more than for other specialties.

Interview 2

because psych(iatry) can be so complex; I feel like it might be hard for the family doc[tor] to know when to refer or what’s appropriate to refer.

Interview 3

#### Applicability of Electronic Consultation Would Be Higher if Region-Specific Advice Was Provided and Integrated With Access to Other Services

Specifically, in the case of mental health care, PCPs suggested that e-consults should be provided by psychiatrists in the same jurisdiction or geographic area to ensure common knowledge about “the standard of care or the resources or the types of patients or just the way the system works...” (Interview 2). The rural PCPs also preferred the same, but they had been accustomed to accessing specialists through telemedicine. Many instances wherein e-consult could be integrated with other services were mentioned, including phone consultation and in-person assessment. Experienced PCPs particularly expressed an interest in phone consultation:

I think, actually, an email saying, can we find a time to chat, could be very useful...

Interview 1

Similarly, face-to-face consultation was not considered to be well-integrated with e-consult. In one example, a psychiatrist e-consultant offered to see a rural patient in-person, but the patient had to travel for 6 hours to meet the consultant.

Rather than a consult, the PCPs viewed e-consult as a potential tool for shared care of patients by functioning as means of communication between providers to facilitate “collaboration” rather than “consultation” to bring providers “together with the best plan” (Interview 1). The participants discussed a preference to be consulting a psychiatrist who has knowledge of the patient and ideally has met them previously:

I think the main thing that is helpful around an e-consult, the way it helps me quickly, if it’s somebody that they already know, and I don’t have to go through the whole history, et cetera...

Interview 1

However, a barrier for this type of care is an appropriate compensation model. As one PCP described:

...although we’re trying to advance care, and be more streamlined, and be more efficient, and work together in a more collaborative model, and meet patients where they’re at, et cetera, we’re not getting paid for this approach.

Interview 1

#### New Primary Care Providers and Primary Care Providers From Rural Areas May be More Ready to Adopt Electronic Consultation

Experienced PCPs clearly preferred verbal communication with consultants:

A practical issue for me is I think I’m more eloquent when I speak to somebody, than when I try to type.

Interview 1

Conversely, new PCPs were more open to using e-communication and were more optimistic about future applications of e-consults:

I think this has a good potential to be a light touch, umm...kind of intervention that can have a meaningful impact...

Interview 2

PCPs from rural areas were focused on gaining access and improving patient experience, and were already accustomed to using telemedicine for specialist care; thus, they were more acceptable of e-consult:

...for most of our patients, you can get a fairly timely response from e-consult, and certainly it provides the support that I need to help manage my patient without having them drive anywhere.

Interview 4

In fact, e-consult was seen as a necessary tool for access in the constrained mental health system:

I don’t have a choice really to use the e-consult because a lot of time it takes just too long to see the psychiatrist.

Interview 4

### Recommendations

For the most part, the quantitative and qualitative data converged and could be interpreted to yield 5 recommendations for future uptake and expansion of e-consult between PCPs and psychiatrists.

First, PCPs can be educated regarding the feasibility and promptness of e-consult in receiving psychiatric advice. They should also be given examples regarding the types of requests that can be addressed using e-consult.

Second, the primary use for psychiatric e-consult is mostly for seeking pharmacological advice. Unless specified before, this may cover any and all psychiatric disorders and medication classes. The e-consult service needs to be adequately staffed with questions appropriately directed to the most knowledgeable specialists.

Third, PCPs, who are new to practice and who are from rural areas, may be more receptive to psychiatric e-consult, but not exclusively, as long wait times for specialist care are universal.

Fourth, in mental health care, where community services and social determinants of health are important, e-consult psychiatrists ideally must have familiarity or relationships with the communities, and wherever possible with the providers, that their patients consult to.

Fifth, given the potential nuances of the psychiatric patient, e-consult should be integrated with other psychiatric services including telephone consultation and face-to-face assessment either in-person or by telemedicine, as well as with methods of communicating for ongoing collaboration.

## Discussion

### Summary of Findings

E-consult for psychiatry accounted for 4% of all e-consults on the 2 platforms that we studied, and represented nearly all psychiatric diagnostic categories. Most e-consults were addressed in a single exchange and completed within 24 hours. The quantitative and qualitative data converged to yield recommendations for the implementation, integration, and staffing of the psychiatry e-consult service to optimize utility and uptake.

### Comparisons With Previous Work

Our finding that 4% of all e-consults were for psychiatry is strikingly similar to the findings in previous reports [6]. This percentage represents underutilization of e-consults, given the high proportion of individuals with mental illness who seek care in primary practice [14,15]. In our qualitative data, we found that PCPs perceived that e-consult had limited utility in psychiatric issues, which were considered more nuanced, and preference was given to face-to-face assessment of these patients. Similar to this finding, a US study reported a recommendation for an in-person assessment in a quarter of psychiatry e-consults [18], and another US study found a higher rate of conversion to face-to-face visits for psychiatry e-consults than other medical specialties [13]. These findings support that e-consult is not a complete solution but could and should facilitate stepped care approaches to ensure access to the right care at the right time. We observed that the most common actual and suggested use for psychiatry e-consult was medication advice, consistent with the findings of another descriptive study of psychiatry e-consults [18]. We also found that PCPs who are from rural areas and those who are new to practice are more likely to be receptive to using psychiatry e-consult, reflecting differential adoption of technology and program change based on geography, age, and experience [19]. Ultimately, many of the recommendations that we arrived at are applicable to e-consult more broadly and have been described by others disciplines, such as expansion to be a part of the triage and referral pathway [5,20], and for comanagement [5].

### Limitations

A limitation of this study was that data were collected too early during the implementation of both the e-consult platforms, which may not be generalizable to more established services or services that function in different ways. Our sample sizes were not large, but the volumes of e-consults were similar to the volumes that have been reported for other platforms during their early use [6,18]. Furthermore, for generating recommendations regarding future directions for psychiatry e-consult, we feel that the quantitative data were sufficiently informative, and that the themes from the qualitative interviews were saturated and triangulated well with the existing literature [6,13,19] and our personal experience with continued use of e-consult. By maximum variation sampling, we were able to identify pertinent provider differences amidst the common themes. Although we reported on the content of psychiatrist’s response, we did not assess the quality of these responses or the actual rate at which recommendations were followed by the PCPs.

### Conclusions

For health care technology, a key facilitator of adoption is the proof of utility [19]. We specifically undertook this study due to the issues of patient complexity and PCP skepticism that we encountered with respect to e-consultation for psychiatry. Empirical data that support the benefits of e-consult are emerging, and our study has identified some factors that could be optimized for improving the utility and the uptake of e-consults between PCPs and psychiatrists. Within current resource-constrained environments, new models of integrated care for mental illness are needed to improve quality of care for patients. Although overall access to psychiatry needs to be improved [21], the psychiatrist who specializes in complex mental health problems can typically offer advice with an e-consult to PCPs, and enhance their feeling of support while caring for their patients [12]. Additional study of the impact on patient outcomes and costs is required, along with established characteristics of a good e-consult psychiatrist and features of an effective e-consult. Resource considerations are essential as compensation and practice models are not often well aligned to incentivize novel methods of communication and collaboration.

## Abbreviations

e-consult: electronic consultation
PCP: primary care provider

## References

[1] Kreindler SA (2010). Policy strategies to reduce waits for elective care: a synthesis of international evidence. Br Med Bull.

[2] Berthelot J, Sanmartin C (2006). http://www.statcan.gc.ca/pub/82-575-x/82-575-x2006002-eng.htm.

[3] Barua B (2015). https://www.fraserinstitute.org/studies/waiting-your-turn-wait-times-for-health-care-in-canada-2015-report.

[4] Vimalananda VG, Gupte G, Seraj SM, Orlander J, Berlowitz D, Fincke BG, Simon SR (2015). Electronic consultations (e-consults) to improve access to specialty care: a systematic review and narrative synthesis. J Telemed Telecare.

[5] Keely E, Liddy C, Afkham A (2013). Utilization, benefits, and impact of an e-consultation service across diverse specialties and primary care providers. Telemed J E Health.

[6] Liddy C, Afkham A, Drosinis P, Joschko J, Keely E (2015). Impact of and satisfaction with a new eConsult service: a mixed methods study of primary care providers. J Am Board Fam Med.

[7] Jaakkimainen L, Glazier R, Barnsley J, Salkeld E, Lu H, Tu K (2014). Waiting to see the specialist: patient and provider characteristics of wait times from primary to specialty care. BMC Fam Pract.

[8] Goldner EM, Jones W, Fang ML (2011). Access to and waiting time for psychiatrist services in a Canadian urban area: a study in real time. Can J Psychiatry.

[9] Malowney M, Keltz S, Fischer D, Boyd JW (2015). Availability of outpatient care from psychiatrists: a simulated-patient study in three U.S. cities. Psychiatr Serv.

[10] Lim KL, Jacobs P, Ohinmaa A, Schopflocher D, Dewa CS (2008). A new population-based measure of the economic burden of mental illness in Canada. Chronic Dis Can.

[11] (2016). Mental Health Commission of Canada.

[12] Golberstein E, Kolvenbach S, Carruthers H, Druss B, Goering P (2017). Effects of electronic psychiatric consultations on primary care provider perceptions of mental health care: survey results from a randomized evaluation. Healthc (Amst).

[13] North F, Uthke LD, Tulledge-Scheitel SM (2015). Internal e-consultations in an integrated multispecialty practice: a retrospective review of use, content, and outcomes. J Telemed Telecare.

[14] Lesage AD, Goering P, Lin E (1997). Family physicians and the mental health system. Report from the Mental Health Supplement to the Ontario Health Survey. Can Fam Physician.

[15] (2014). Centers for Disease Prevention and Control.

[16] Shannon-Baker P (2016). Making paradigms meaningful in mixed methods research. J Mix Methods Res.

[17] Creswell JW (2013). http://digitalcommons.unl.edu/cgi/viewcontent.cgi?article=1047&context=dberspeakers;.

[18] Lowenstein M, Bamgbose O, Gleason N, Feldman MD (2017). Psychiatric consultation at your fingertips: descriptive analysis of electronic consultation from primary care to psychiatry. J Med Internet Res.

[19] de Grood C, Raissi A, Kwon Y, Santana MJ (2016). Adoption of e-health technology by physicians: a scoping review. J Multidiscip Healthc.

[20] Patterson V, Humphreys J, Chua R (2004). Email triage of new neurological outpatient referrals from general practice. J Neurol Neurosurg Psychiatry.

[21] Clatney L, Macdonald H, Shah SM (2008). Mental health care in the primary care setting: family physicians' perspectives. Can Fam Physician.

